# Water aerobics in pregnancy: cardiovascular response, labor and neonatal outcomes

**DOI:** 10.1186/1742-4755-5-10

**Published:** 2008-11-21

**Authors:** Erica P Baciuk, Rosa I Pereira, Jose G Cecatti, Angelica F Braga, Sergio R Cavalcante

**Affiliations:** 1 Department of Obstetrics and Gynecology, School of Medical Sciences, University of Campinas (UNICAMP), Sao Paulo, Brazil; 2 Department of Anesthesiology, School of Medical Sciences, University of Campinas (UNICAMP), Sao Paulo, Brazil

## Abstract

**Background:**

To evaluate the association between water aerobics, maternal cardiovascular capacity during pregnancy, labor and neonatal outcomes.

**Methods:**

A randomized, controlled clinical trial was carried out in which 34 pregnant women were allocated to a water aerobics group and 37 to a control group. All women were submitted to submaximal ergometric tests on a treadmill at 19, 25 and 35 weeks of pregnancy and were followed up until delivery. Oxygen consumption (VO_2 max_), cardiac output (CO), physical fitness, skin temperature, data on labor and delivery, and neonate outcomes were evaluated. Frequency distributions of the baseline variables of both groups were initially performed and then analysis of the outcomes was carried out. Categorical data were compared using the chi-square test, and numerical using Student's t or Mann-Whitney tests. Wilk's Lambda or Friedman's analysis of repeat measurements were applied for comparison of physical capacity, cardiovascular outcomes and maternal temperature.

**Results:**

VO_2 max _and physical fitness were higher in both groups in the second trimester, returning to basal levels in the third trimester. In both groups, CO increased as pregnancy progressed and peak exercise temperature was higher than resting temperature, increasing further after five minutes of recovery and remaining at this level until 15 minutes after exercise completion. There was no difference between the two groups regarding duration (457.9 ± SD 249.6 vs 428.9 ± SD 203.2 minutes) or type of delivery. Labor analgesia was requested by significantly fewer women in the water aerobics group (27% vs 65%; RR = 0.42 95%CI 0.23–0.77). Neonatal results were similar in both groups.

**Conclusion:**

The regular practice of moderate water aerobics by sedentary and low risk pregnant women was not detrimental to the health of the mother or the child. There was no influence on maternal cardiovascular capacity, duration of labor or type of delivery; however, there were fewer requests for analgesia during labor in the water aerobics group.

## Background

Women's lifestyles and their repercussions in pregnancy and delivery have been the subject of much debate, the principal concerns being that exercise may interfere with fetal-placental demands, increasing the risk of teratogenic abnormalities or of compromising fetal development or growth [1].

Today, women are encouraged to practice regular physical activity as part of a healthy lifestyle. Many women already practice regular aerobic conditioning or strength-building exercise prior to becoming pregnant. Others view pregnancy as an opportunity to change their lifestyle by introducing more healthy habits. However, physicians have traditionally counseled their patients who practice exercise to reduce exercise levels during pregnancy and have discouraged sedentary women from initiating an exercise program at this time [2].

If there are still doubts on the safety of practicing general physical activity during pregnancy, there is also a common recognition that the activity performed in water is safer. Exercise in water promotes a redistribution of body fluids that leads to an increase in central blood volume and cardiac output, mainly due to a secondary increase in stroke volume. On the other hand, blood pressure and heart rate decrease. However, these changes do not affect any parameter of fetal well-being [3].

Recommendations such as those of the American College of Obstetricians and Gynecologists (ACOG) defend the practice of regular, moderate physical activity even for sedentary expectant mothers or for those with slight complications such as gestational diabetes. Absolute and relative contraindications to the practice of exercise must, nevertheless, be respected, including for instance multiple gestation, higher risk of premature labor, incompetent cervix, bleeding during pregnancy, severe heart diseases, fetal growth restriction, and others [4].

Common sense indicates that high intensity activities and/or high impact activities should be avoided since the practice of exercises such as these may expose the mother and fetus to unnecessary risks such as mechanical trauma, restricted development or prematurity [4]. On the other hand, moderate exercise in water presents several advantages since it does not overload the muscular-skeletal structure, reduces edema and prevents an increase in maternal skin temperature [1]. In other words, the ideal heart rate calculated for the intensity of the exercise should be adapted to 60 to 90% of one's age predicted maximum heart rate [2,4]. The use of RPE (Ratings of Perceived Exertion) is recommended in both the American [1] and Canadian [2] guidelines for the intensity evaluation during pregnancy. In fact, Artal & Toole [1] states that target heart rates cannot be used to monitor exercise intensity in pregnancy due to variability in maternal heart rate responses to exercise. Davies et al. [2] suggests the use of a modified version of the conventional age-corrected heart rate target zone but also the use of Borg's scale is suggested.

Reports in the literature show that the interrelationship between physical exercise and pregnancy is complex. The results of the published studies are controversial, and there are few randomized, controlled clinical trials that evaluate the effects of moderate physical activity in water on pregnancy and delivery [5-7]. A recent randomized controlled study showed no differences regarding gestational ages between women practicing or not regular exercises during pregnancy [8].

The objective of this study was to evaluate the association between the practice of water aerobics during pregnancy and maternal cardiovascular capacity, experience at delivery and neonatal outcomes among low risk pregnant women who performed water aerobics compared to those without exercise.

## Methods

### Participants and Design

Pregnant women of < 20 weeks of pregnancy, who were carrying a single fetus, had no gestational risk factors, were receiving prenatal care at this institution and intended to give birth there, were admitted to a randomized, controlled clinical trial. The expectant mothers were provided with information on the objectives of the study, as well as information regarding the possible benefits of practicing exercises during pregnancy, evaluations and procedures that would be carried out in the research protocol. Those who agreed to participate gave their signed, informed consent and answered a questionnaire designed to evaluate their physical fitness at admission. The principles of the Helsinki Declaration were upheld and the research project received the approval of the IRB of the institution (Woman's Hospital of the School of Medical Sciences from the University of Campinas in Brazil) prior to initiation.

Exclusion criteria comprised: to practice regular physical exercise; to have had two or more Cesarean sections, clinical and/or laboratory diagnoses of neurological, cardiovascular, pulmonary, muscular-skeletal or endocrine disorders, and to have any disorder that could represent a risk to the woman's health, such as morbid obesity, severe anemia or vaginal bleeding during pregnancy.

Volunteers were enrolled sequentially and randomized to one of the two study groups. Each sequential number corresponded to a sealed opaque envelope containing the information on the randomization group, according to a previously prepared computer-generated randomization list of numbers, in order to guarantee the concealment. Water aerobic group was the one in which participants would practice exercise, while Control group comprised women who would not carry out any regular physical activity during the entire pregnancy.

The intervention was the regular, moderate practice of water aerobics for 50 minutes three times a week in an indoor swimming pool with water warmed at 28–30°C. Water aerobics was initiated following the first physical evaluation and continued up to delivery. The moderate intensity of exercises during the sessions was assured by monitoring patients' heart rate using a heart rate monitor [2] and kept around 70% of one's predicted maximun heart rate [4].

### Evaluations

All pregnant women were submitted to three physical evaluations during pregnancy: a control evaluation (prior to initiating the practice of water aerobics – at 18–20 weeks of pregnancy); in the second trimester of pregnancy (between 22 and 26 weeks), and in the third trimester of pregnancy (between 32 and 36 weeks).

Maternal cardiovascular capacity was evaluated by a submaximal endurance test, according to Bruce's modified protocol II [9], a multistage treadmill test of graded exercise. The test starts at 2.7 km/hour with no gradient. After three minutes the inclination of treadmill increases to 10% in the same speed. At three minute intervals the gradient is increased by 2% and the speed to 4 and 5.5 km/hour. The mean time for the patient to reach exhaustion point is estimated to be 12 minutes. Prior to each test, weight, height and women' vital signs (heart rate, resting systolic and diastolic blood pressure) and fetal heart rate were recorded. Next, the pregnant woman was placed on an ergometric treadmill to record basal or control data: ECG print-out, heart rate, blood pressure and maternal skin temperature. During the ergometric test, women were monitored using an integrated computerized ergometric system (APEX TEB 2200) and submitted to the protocol described above.

The test was designed to stop if one of the following events occurred: exhaustion, significant alterations in heart rate, electrocardiogram or skin temperature, or difficulty in accompanying the set speed without running. After completing the ergometric test, the woman was requested to walk for 3 minutes at a speed of 2.7 km/hour at 0% elevation so as not to cease the exercise abruptly.

Blood pressure was measured by the auscultatory method at the end of each stage of exercise and up to 6 minutes following exercise, using a mercury column sphygmomanometer. Skin temperature, in °C, was measured prior to exercise (at rest), at peak exercise, and at 5, 10 and 15 minutes following completion of exercise (recovery), using a digital cutaneous thermometer. Fetal heart rate was monitored using a portable fetal heart monitor (DF-25 Medcir) at rest, and at 10 and 15 minutes of recovery. Endurance tests were always carried out at the same time of the day, in a cool, well-ventilated room.

The study investigators were informed when any woman was hospitalized for delivery, and were present to follow up the labor and delivery and to collect data. The medical team that provided care during delivery had no knowledge of the randomization group of the individual patient.

Baseline variables were: maternal age, body weight, pre-gestational body mass index (BMI) (it was estimated dividing the pre-gestational weight value informed by the woman by the square height), parity, previous abortions and Caesarean sections, and maternal education level.

### Outcome measures

Maternal-dependent variables were defined as: cardiovascular capacity and skin temperature. Cardiovascular capacity was evaluated by the maximum oxygen consumption (VO_2_), by cardiac output in L.min^-1 ^and by the metabolic equivalent (MET). The equation for VO_2 _(ml/kg/min) is speed × [0.1 + (gradient/100 × 1.8)] + 3.5 and all these parameters were directly obtained from the integrated computerized system used. Secondary variables were heart rate (HR), in beats per minute and blood pressure in mmHg. Fetal-dependent variable was fetal heart rate (FHR).

Variables regarding labor and delivery that were considered were: the woman's request for analgesia, cervical dilation at the time of analgesia indication, length of labor and types of delivery. Cervical dilation (cm) at analgesia indication was measured by the obstetrician at the time at which analgesia was given. The duration of labor (minutes) was calculated from the onset of regular uterine contractions to delivery and was obtained from the delivery records. Types of delivery were defined as spontaneous vaginal delivery, forceps delivery, or Cesarean section.

Dependent variables for the newborn were obtained from information on the obstetrics and neonatal records: birth weight in grams, gestational age in weeks at delivery, and vitality of the newborn, defined as the set of organic functions of the newborn evaluated at the first and fifth minutes of life according to the Apgar Score.

Discontinuation criteria were defined as: any change that could put the health of the mother or the fetus at risk; irregular prenatal care; or the woman giving up the water aerobics sessions or any evaluation, but their data were included in the final analysis because an intention-to-treat analysis approach was chosen.

### Analysis

Calculation of sample size was based on the difference between the mean VO_2 _max and cardiac output measured at two different moments in the control and water aerobics groups in a study carried out by Prevedel et al. [5]. For the comparison of initial and final intra-group variables, a sample size of 12 would be sufficient, and for the inter-group comparison of variables, a sample size of 30 in each group would be required [10,11]. Sample size was increased by around 20% to compensate for subject non-compliance or for lost-to-follow-up, making a total of 71 expectant mothers, with a power of 80% and a type I error of 0.05.

The information collected on the case report forms was transferred to a database using the Epi Info software program, version 3.2.2. Frequency distributions of the baseline variables of both groups were analyzed to verify their comparability and to exclude confounding factors. Analysis of the dependent variables was then carried out in both groups.

In the bivariate analysis, the qualitative variables were compared using the chi-square test, and the numerical variables using Student's t-test or the Mann-Whitney test. Wilk's Lambda, corresponding to the multivariate analysis of variance (MANOVA), was used for the comparison of means of numerical variables with repeat measurements or, in the case of non-normal data, Friedman's analysis of repeat measurements was applied. The Epi Info software program, version 3.2.2 and the SAS program, version 8.2 were used in all analysis procedures.

## Results

### Participants

**Figure 1:**
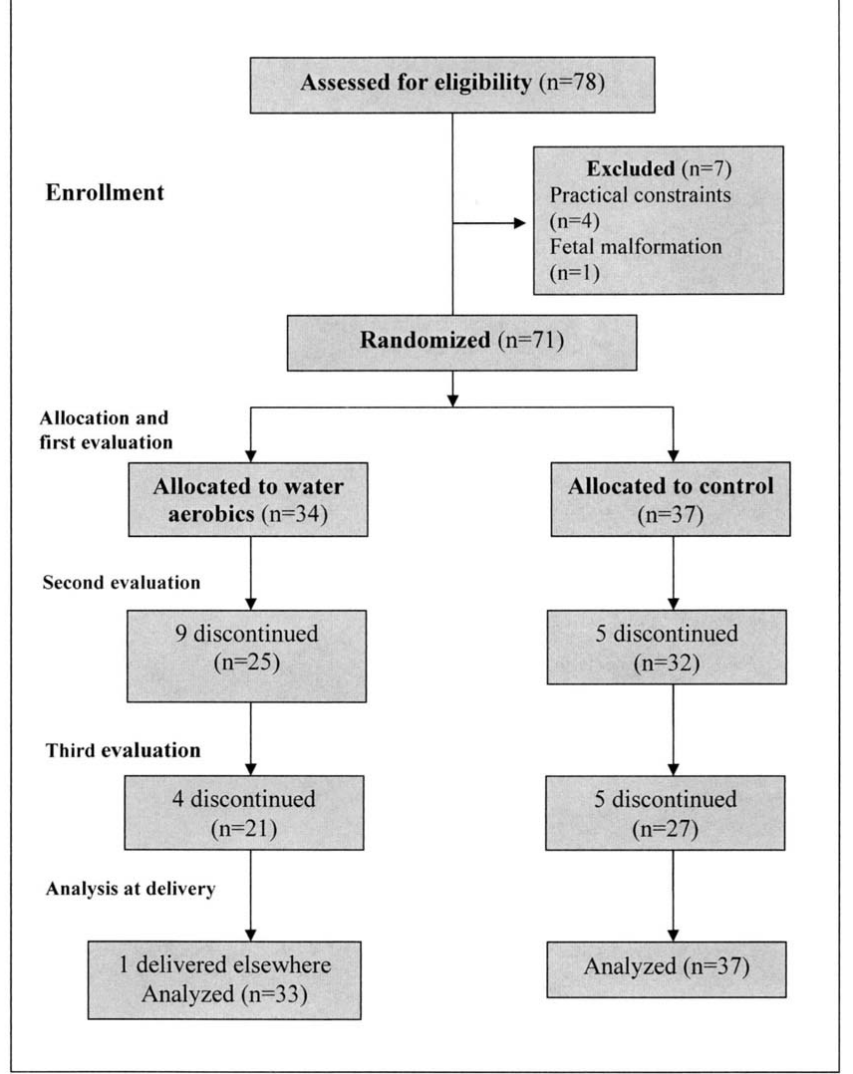
Flow chart of the study.

Of the 78 expectant mothers eligible for inclusion in this trial, 34 were randomized to the water aerobics group and 37 to the control group, while 7 women were excluded before randomization, one because of morbid obesity, two because admission ultrasonography revealed abnormalities (one fetal malformation and one fetal death) and four because they considered too difficult to follow the program of water aerobics (Figure 1). Baseline characteristics of the 71 participants are shown in Table 1. Physical evaluations were carried out on average at: 19 weeks (first), 25 weeks (second) and at 35 weeks of pregnancy (third).

**Table 1:** Clinical characteristics of expectant mothers according to group.

**Variables**	**Water Aerobics**	**Control**
	n = 34	n = 37
Age (years)	25.8 ± 4.6	24.4 ± 5.8
Weight (kg)	63.8 ± 12.7	60.8 ± 10.2
Pre-gestational BMI	24.1 ± 4.5	23.4 ± 3.8
% of nullipara	47.1 (16)	62.2 (23)
% with previous abortions	20.6 (7)	10.8 (4)
% with previous C section	11.8 (4)	24.3 (9)
% with only primary school education	47.1 (16)	27.0 (10)

Continuous data are expressed by means ± standard deviation.

Differences not statistically significant using Student's t or χ^2 ^tests as appropriate

### Maternal outcomes

**Figure 2:**
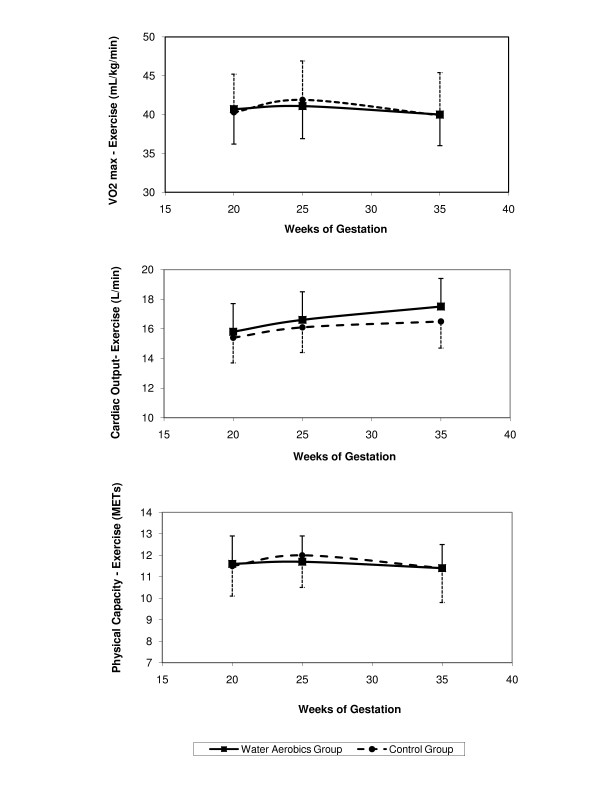
VO_2_max, cardiac output and metabolic equivalent at the three evaluation moments, according to group.

The indicators of maternal cardiovascular capacity were similar in the two groups throughout pregnancy (Figure 2). VO_2 _max and physical capacity (MET) were greater during the second trimester; returning, however, in the third trimester to values similar to those measured at the beginning of pregnancy. Cardiac output increased as pregnancy progressed.

**Figure 3:**
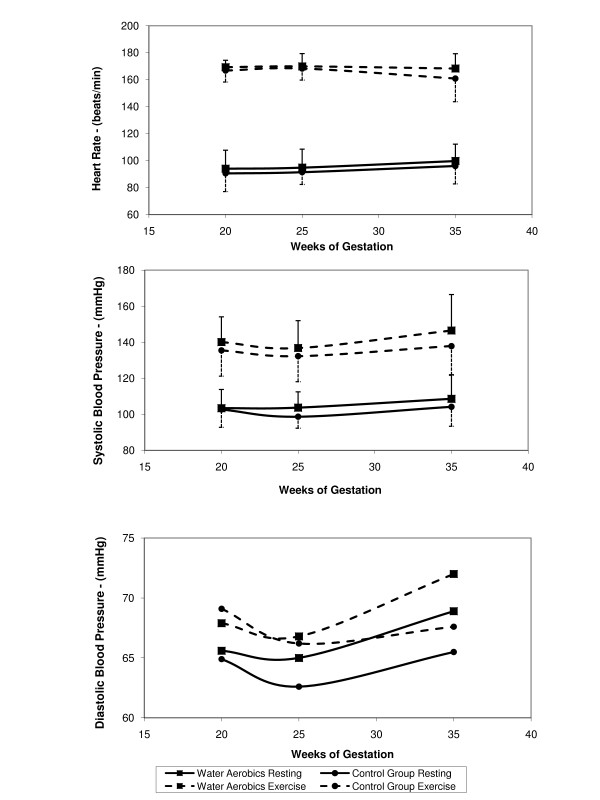
Heart rate, systolic and diastolic pressure at the three evaluation moments, according to group.

HR was also similar in both groups. HR measured at rest showed a significant increase from the second to the third trimester. HR measured during exercise decreased from the second to the third trimester of pregnancy, reaching values lower than those registered at the first evaluation (Figure 3).

Systolic blood pressure showed time and group effects. In the water aerobics group, systolic blood pressure remained unaltered from the first to the second trimester of pregnancy and increased in the third, while in the group of control women, first there was a reduction in values, followed by an increase. Peak exercise systolic blood pressure showed lower values at the second evaluation and increased at the third evaluation, returning to values close to rest (Figure 3).

Diastolic blood pressure was similar throughout pregnancy in the two groups. There was a marginal reduction in resting measurements between the first and the second evaluations and an increase at the third evaluation. Peak exercise diastolic blood pressure behaved similarly to peak exercise systolic pressure (Figure 3).

**Figure 4:**
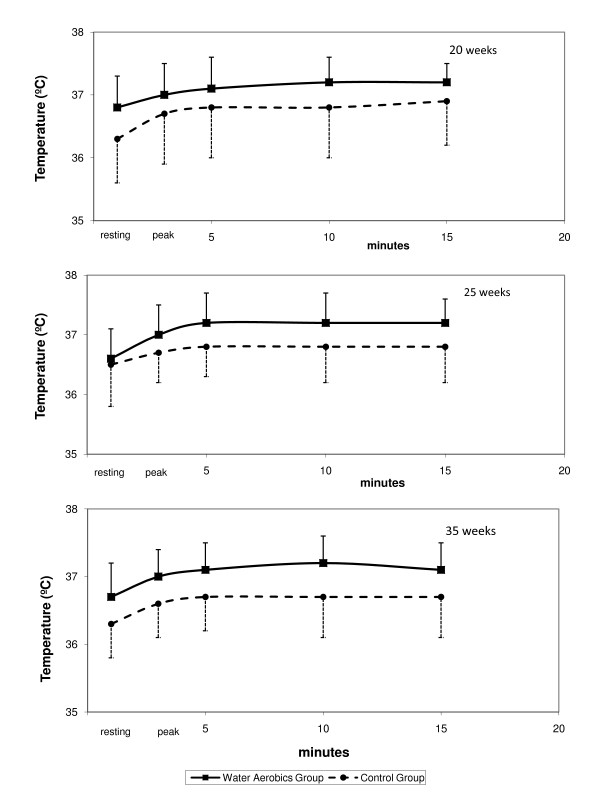
Maternal skin temperature at the three evaluation moments, according to group.

There were no statistically significant differences in maternal skin temperature in either group over the three evaluations. Peak exercise values were greater than resting values, increasing at 5 minutes of recovery and remaining unaltered up to 15 minutes of post exercise recovery (Figure 4).

### Labor outcome

There were no statistically significant differences in the duration of labor between the two groups or in the type of delivery. However, significantly fewer women in water aerobics group (27.3%) than in control group (64.9%) requested analgesia, which was initiated in women of both groups at approximately 6 – 7 cm of cervical dilation (Table 2). This represented a 58% reduction in the risk of having analgesia requested (RR = 0.42; 95%CI 0.23–0.77). Even controlling for parity and level of schooling, this difference in requesting analgesia remained highly significant (data not shown in table).

**Table 2:** Characteristics of labor, delivery and neonatal outcomes according to group.

**Variables**	**Water aerobics**	**Control**	**P**
Request for analgesia – n (%)	9 (27.3)	24 (64.9)	0.004**
Length of labor (min)^a^	457.9 ± 249.6	428.9 ± 203.2	0.69*
C-section – n (%)	12 (36.4)	17 (45.9)	0.57**
Birth weight (g)	3222.2 ± 562.7	3312.7 ± 656.1	0.54*
Apgar Score 1^st ^minute ≥ 7 (%)	97	94.6	0.54**
Gestational age (weeks)	39.2 ± 2.2	39.1 ± 1.6	0.73*

Continuous data are expressed by means ± standard deviation.

* Student's t-test ** χ^2 ^Yates ^a ^Only for vaginal deliveries

### Fetal and neonatal outcomes

**Figure 5:**
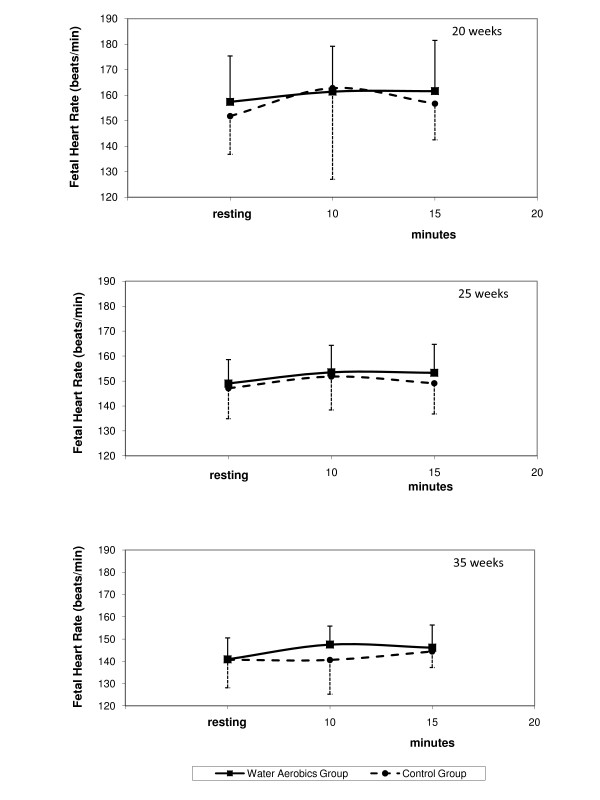
Fetal heart rate at the three evaluation moments, according to group.

There was no statistically significant difference in pre-exercise FHR in the water aerobics group compared to the control group, and values decreased as the pregnancies evolved. FHR increased during maternal exercise at the first and second evaluations and remained at this level at 10 and 15 minutes following completion of exercise in both groups. In the third trimester, a difference was observed between the groups. In the water aerobics group, FHR increased at 10 minutes, decreasing at 15 minutes of recovery. In the control group, FHR increased only at 15 minutes following completion of maternal exercise (Figure 5).

Neonatal results were similar in both groups and are also shown in Table 2. Mean birth weight was 3.222 grams and mean gestational age was 39 weeks for water aerobics group while respectively 3312.7 g and 39.1 weeks for control group. The majority of newborns obtained Apgar scores ≥ 7 in the first minute in both groups, while all of them obtained scores ≥ 7 at the fifth minute.

## Discussion

The current study found that there was no effect on the cardiovascular capacity of the women or on the duration of labor or the type of delivery for women practicing water aerobics regularly during pregnancy in comparison to those not practicing exercise at all. However, fewer women in the exercise group requested analgesia.

Most of the knowledge available today on the practice of physical exercise during pregnancy is based on longitudinal and, mainly, observational studies. This randomized controlled trial may contribute towards greater comprehension of the interaction between pregnancy and the practice of water aerobics, as well as the repercussions of exercise in labor and on the well-being of the newborn infant.

Cardiovascular parameters of VO_2 _max, cardiac output and submaximal endurance heart rate showed similar results in both groups, showing that the practice of water aerobics according to the regimen described in this study failed to have any effect on physical capacity. These results are in agreement with data published by Prevedel et al. [6].

VO_2 _max and physical capacity (MET) increased during the second trimester of pregnancy and returned to previous values during the third for both groups, while Wolfe et al [12] reported an increase in VO_2 _max with the progression of pregnancy in women who participated in a program of physical conditioning, while values remained unchanged in a group of sedentary pregnant women, but evaluations were made using ergometric bicycles. Santos et al. [13] reported a substantially increase in submaximal exercise capacity in overweight pregnant women who were submitted to aerobic exercise sessions also in a randomized controlled trial.

The increase in resting heart rate over the course of pregnancy was an expected result and may be explained by a reduction in vagal parasympathetic control. The smaller increase in heart rate during exercise in the third trimester of pregnancy may be a result of the reduction in the response to sympathetic stimulation during pregnancy [14,15].

The maintenance or reduction of systolic blood pressure in the second trimester and reduction of diastolic blood pressure confirm that the response of arterial blood pressure during pregnancy is related to a reduction in peripheral vascular resistance [16-18]. At the second evaluation, the lower value of diastolic blood pressure during exercise may be a result of the lower response to sympathetic stimulation. The expected effect of vascular pumping caused by the balance between vasoconstriction of the non-active musculature versus vasodilatation of the active musculature may not be sufficiently adapted to overcome the present reduction in peripheral vascular resistance. This preloading reduction may consequently lead to a post-loading reduction and lower systolic blood pressure, which was probably compensated by an increase in the ventricular contractility during exercise at the beginning of pregnancy [18,19]. The rise in systolic blood pressure during exercise in the third trimester showed an adequate adjustment in the pressure-volume curve at acute endurance.

These results show that the pregnant women had an adequate adaptive response to the demands of a normal pregnancy. Moreover, they were capable of compensating for greater demands such as an endurance test or the practice of regular, moderate physical activity in water despite the fact that no effect was obtained on cardiovascular conditioning during pregnancy.

The reduction in fetal heart rate with the progression of pregnancy may be directly related to the immaturity, particularly parasympathetic immaturity, of the autonomous fetal nervous system in the first half of pregnancy. The increase in FRH observed during recovery may be explained by the liberation of maternal catecholamines during physical exercise, confirmed by the increase in maternal HR [20]. However, in the third trimester, the group that practiced water aerobics appeared to show a more adequate autonomic response than the control group. Probably this response accompanies the reduction in the maternal response to sympathetic stimulation during this period of pregnancy [15,18].

Van Doorn et al. [21] described an increase in FHR five minutes after maternal peak exercise. Veille et al. [22]) reported no change in FHR during 30 minutes following moderate maternal exercise. Carpenter et al. [23] also failed to find any significant differences in FHR at rest and 5 minutes following maternal peak exercise.

To avoid possible side effects of maternal-fetal hyperthermia, endurance tests were always carried out at the same time of the day, in the same environmental conditions and in a cool, well-ventilated room, in order to minimize the increase in skin temperature of the volunteers. The acceptable maternal skin temperature of approximately 38.9°C was respected [24].

Maternal temperature increased in response to strenuous exercise and remained high in both groups until 15 minutes following the end of exercise, causing no ill effects to fetal vitality as can be seen from the response in FHR. These results are in agreement with data published by Soultanakis-Aligianni [24] who described an increase in maternal temperature during exercise of approximately 0.7°C and 0.4°C at 20 and 32 weeks of pregnancy, respectively. This increase found in skin temperature was lower for the cases studied by Larson & Lindqvist [25] and Lindqvist et al.[26] and one possible explanation refers to the fact that these authors measured the core temperature instead skin temperature. The difference between skin and core temperature might be interpreted as a safety mechanism during pregnancy exercise. This would give a possible explanation to the trend towards increased skin temperature in the exercise group.

Some limitations of the current study could be pointed out. Probably the most important refers to the practical difficulty of maintaining a high compliance with the water aerobics program. Although these women had free access to the swimming pool and professional oriented sessions of water aerobics, plus the costs for transport three times per week, around one third of them discontinued the program during pregnancy due to logistic and family constraints, including job restrictions, care of children and home affairs.

The results of this study show that the regular practice of moderate water aerobics during pregnancy by low risk women who were previously sedentary is not detrimental to the health of the mother or the child. Although there was no effect on the cardiovascular capacity of the expectant mothers, on the duration of labor or the type of delivery, fewer women in the water aerobics group requested analgesia, probably because of better psycho-physical condition. Clap III [27], who studied women that practiced physical activity and who either continued or spontaneously stopped exercising (control group) in the first trimester of pregnancy, observed similar results. This author observed a lower incidence of Cesarean sections, shorter duration of labor, a greater number of vaginal deliveries, and less need for epidural anesthesia among women who continued exercising during pregnancy. Moreover, infants born to women in the exercise group were smaller and had higher Apgar scores at the first minute.

Neonatal results from this present study confirm the wellbeing of the newborn infants born to mothers who initiated regular physical activity in water during pregnancy. Therefore this kind of exercise could be recommended to mothers willing to practice any physical activity during pregnancy [27]. The babies had adequate weight, gestational age and vitality at birth, confirming the trend that already exists in the literature that moderate, regular physical activity has no influence on prematurity or on the weight of the newborn infant. However, the adequacy of the exercise has to be assured since the practice of physical activity that is rigorous either in its intensity, duration or frequency is associated with low neonatal birthweight [5,28-33].

## Conclusion

The regular practice of moderate water aerobics by low risk and previously sedentary expectant mothers offers no risk to the health of the mother or the child. Although there was no effect on the cardiovascular capacity of the women or on the duration of labor or the type of delivery, fewer women in the exercise group requested analgesia.

## Abbreviations

ACOG: American College of Obstetricians and Gynecologists; BMI: body mass index; CO: cardiac output; ECG: electrocardiogram; FHR: fetal heart rate; HR: heart rate; MANOVA: multivariate analysis of variance; MET: metabolic equivalent; RR: Risk ratio; VO_2 max_: oxygen consumption.

## Competing interests

The authors declare that they have no competing interests.

## Authors' contributions

EPB, RIP and JGC participated in all steps of the study, including research planning, data collection, analysis and writing the manuscript. AFB participated in the project planning and review of the manuscript. SRC participated in data collection and review of the manuscript. All authors gave suggestions, read the manuscript carefully, fully agreed on its content and approved its final version.
